# Complete Genome Assembly of a Quality Control Reference Isolate, *Moraxella catarrhalis* Strain ATCC 25240

**DOI:** 10.1128/genomeA.00938-14

**Published:** 2014-09-18

**Authors:** H. E. Daligault, K. W. Davenport, T. D. Minogue, K. A. Bishop-Lilly, D. C. Bruce, P. S. Chain, S. R. Coyne, K. G. Frey, J. Jaissle, G. I. Koroleva, J. T. Ladner, C.-C. Lo, L. Meincke, C. Munk, G. F. Palacios, C. L. Redden, S. L. Johnson

**Affiliations:** a Los Alamos National Laboratory, Los Alamos, New Mexico, USA; b Diagnostic Systems Division, USAMRIID, Fort Detrick, Maryland, USA; c Naval Medical Research Center, NMRC-Frederick, Fort Detrick, Maryland, USA; d Henry M. Jackson Foundation, Bethesda, Maryland, USA; e Center for Genome Sciences, USAMRIID, Fort Detrick, Maryland, USA

## Abstract

Generally an opportunistic pathogen in the United States, *Moraxella catarrhalis* has acquired resistance to multiple antibacterial/antimicrobial agents. Here, we present the complete 1.9-Mb genome of *M. catarrhalis* strain ATCC 25240, as deposited in NCBI under the accession number CP008804.

## GENOME ANNOUNCEMENT

*Moraxella catarrhalis*, previously known as *Branhamella catarrhalis*, is the causative agent of many upper respiratory tract infections in the United States. *M. catarrhalis* often exacerbates chronic obstructive pulmonary disease (COPD) and may cause nearly one-fifth of bacterial ear infections in the United States (1–3). Infections are generally limited to children or elderly people; however, the pathogen exhibits high-level β-lactamase resistance, making it a concern for immunocompromised adults (4, 5). To increase the number of reference genomes for diagnostic development and phylogenetic reconstructions (as of this writing only one complete genome is available in public databases), we sequenced and assembled the genome of *M. catarrhalis* strain 25240 into a single closed chromosome (6).

High quality genomic DNA was extracted from a purified isolate using QIAgen Genome Tip-500 at USAMRIID’s Diagnostic Systems Division (DSD). Specifically, a 100-mL bacterial culture was grown to stationary phase and nucleic acid extracted as per the manufacturer’s recommendations. Sequence data were generated using a combination of Illumina and 454 technologies (7, 8). For this genome assembly, we constructed and sequenced an Illumina library of 100-bp reads to high coverage (300-fold genome-coverage) as well as a separate long-insert paired-end library (average insert size 7,431 ± 1,858 bp, run on the Roche 454 Titanium platform to 17-fold genome coverage). The two libraries were assembled together in Newbler (Roche) and the consensus sequences computationally shredded into 2-kbp overlapping fake reads (shreds). The raw reads were also assembled in Velvet and those consensus sequences computationally shredded into 1.5-kbp overlapping shreds (9). Draft data from all platforms were then assembled together with ALLPATHS and the consensus sequences computationally shredded into 10-kbp overlapping shreds (10). We then integrated the Newbler consensus shreds, Velvet consensus shreds, ALLPATHS consensus shreds, and a subset of the long-insert read-pairs using parallel Phrap (High Performance Software, LLC). Possible misassemblies were corrected and some gap closure was accomplished with manual editing in Consed (11–13).

Automatic annotation for the *M. catarrhalis* ATCC 25240 genome utilized an Ergatis-based workflow at LANL with minor manual curation. Annotation located 1,742 coding genes, 50 tRNAs, and 12 rRNAs. The final 1,941,566-bp assembly has 41.5% G+C content. Preliminary review of the annotated genome suggests that over 30 drug resistance genes are present in the genome.

### Nucleotide sequence accession number.

The final sequence has been deposited to GenBank under the accession number CP008804.
